# Cancer Feature Selection and Classification Using a Binary Quantum-Behaved Particle Swarm Optimization and Support Vector Machine

**DOI:** 10.1155/2016/3572705

**Published:** 2016-08-24

**Authors:** Maolong Xi, Jun Sun, Li Liu, Fangyun Fan, Xiaojun Wu

**Affiliations:** ^1^Department of Control Technology, Wuxi Institute of Technology, Wuxi, Jiangsu 214121, China; ^2^Department of Computer Science, Jiangnan University, Wuxi, Jiangsu 214122, China; ^3^Affiliated Hospital of Jiangnan University, Wuxi, Jiangsu 214062, China

## Abstract

This paper focuses on the feature gene selection for cancer classification, which employs an optimization algorithm to select a subset of the genes. We propose a binary quantum-behaved particle swarm optimization (BQPSO) for cancer feature gene selection, coupling support vector machine (SVM) for cancer classification. First, the proposed BQPSO algorithm is described, which is a discretized version of original QPSO for binary 0-1 optimization problems. Then, we present the principle and procedure for cancer feature gene selection and cancer classification based on BQPSO and SVM with leave-one-out cross validation (LOOCV). Finally, the BQPSO coupling SVM (BQPSO/SVM), binary PSO coupling SVM (BPSO/SVM), and genetic algorithm coupling SVM (GA/SVM) are tested for feature gene selection and cancer classification on five microarray data sets, namely, Leukemia, Prostate, Colon, Lung, and Lymphoma. The experimental results show that BQPSO/SVM has significant advantages in accuracy, robustness, and the number of feature genes selected compared with the other two algorithms.

## 1. Introduction

Nowadays, cancer has been one of the most common lethal factors for human beings. Missed and mistaken diagnosis sometimes makes people lose the best chance for appropriate treatments. Therefore, more auxiliary measurements are needed to promote the accuracy of cancer diagnosis and clinical test combined with medical ways [1–4]. With the rapid development of information sciences and molecular biological sciences, gene microarray technology brings people large amount of high-throughput gene profiles which are widely used in cancer diagnosis, clinical inspection, and other aspects. However, microarray expression data are highly redundant and noisy, and most genes are uninformative with respect to studied classes, as only a fraction of genes may present distinct profiles for different classes of samples. As such, effective methods of selecting feature genes for cancer are critically necessary. These methods should be able to robustly identify a subset of informative genes embedded out of a large data set which is contaminated with high dimensional noise.

It was Golub et al. who first employed gene expression data for cancer classification [5]. They proposed to use gene expression data of acute leukemia for cancer classification by adopting “SNR” index to calibrate the contribution of genes to the cancer classification and by using a weighted voting mechanism to distinguish cancer types [5]. This study demonstrated that the use of gene expression data to determine cancer types for the auxiliary medical diagnosis is an effective measure. Afterwards, an increasing number of researchers in the fields of biology and information sciences have proposed many effective feature gene selection methods, so that the research in this discipline is becoming one of the hotspots in bioinformatics.

Currently, there are two categories of the methods of obtaining feature genes for cancer classification based on gene expression data, namely, feature transformation methods and feature selection methods. By definition, feature transformation refers to a way of transforming the original feature attributes into a new set of features that represent the original features to the greatest extent but reduce the dimension as much as possible in order to achieve the purpose of dimension reduction. This means that the new features are low-dimensional features with similar classification abilities. Feature transformation methods for cancer classification by using gene expression data include principal component analysis (PCA) [6], kernel PCA [7], independent component analysis (ICA) values [8], locally linear embedding (LLE) [9], partial least squares (PLS) [10], the maximum margin criterion (MMC) [11], and linear discriminant analysis (LDA) [12]. Conde et al. [13, 14] proposed a feature transform method based on clustering. This approach uses self-organizing tree algorithm to carry out gene clustering and calculates the average gene expression level for each category, which is then accepted as a new feature to establish the cancer classification model. Kan et al. [15] employed PCA to make transformation of the gene expression data of children small round blue cell tumors and then used artificial neural network for classification.

Feature transformation methods can indeed reduce the dimension for gene expression data and can eliminate the “curse of dimensionality” phenomenon due to large number redundant genes so that they can help to establish effective cancer classification models. However, the new features obtained by feature transform property no longer have the original biological meaning; that is, the methods destroy the biological information of the original gene expression data, which makes it impossible to determine the target genes associated with the cancer. For this reason, feature gene selection methods have attracted more attention.

The feature gene selection uses an optimization algorithm to select a subset of the genes, which has the most classification information, from the original gene microarray data. The most commonly used feature gene selection methods can be divided in to filter, wrapper, and embedded ones. Filter algorithm is independent of the subsequent learning algorithm but uses some criteria for scoring gene subsets, which measure the contribution of the genes to classification. Such methods generally use SNR [5], *t* test [16], the correlation coefficient [17], mutual information [18], relief [19], information gain [20], or Fisher discrimination [21]. Obviously, filter methods have advantages such as simplicity, fast calculation, and independence of classification algorithms. However, they evaluate a single gene with some criteria but ignore the correlation between genes, which resulted in a large amount of redundant information contained in candidate genes.

Different from filter methods, wrapper methods combine gene selection and classification method and use training accuracy of the learning algorithm to assess the subset of features to guide gene selection. Such methods include the sequential random search heuristics [22], random forest method [23], and PKLR [24]. In the cancer feature gene selection, a typical wrapper feature selection method combines support vector machine (SVM) and a recursive feature selection method [25]. In this method, support vector machines are used to classify the data set, then each gene is excluded in turn, and the performance change of the SVM after exclusion of the gene is calculated, and afterwards, the gene with the least absolute value of the association weight is removed from the training set until the training set is empty. The gene sets deleted together in the last step are the optimal subset. Li et al. [26] adopted genetic algorithm (GA) to select feature genes of cancer. Zhang et al. [27] coupled a binary particle swarm optimization (BPSO) and the SVM for classification of Colon data set.

Embedded methods are extension of wrapper approaches and undertake feature selection in the process of classifier training, without dividing the data set into a training set and a validation set. Typical embedded algorithms include decision tree [28] and artificial neural networks [29].

In this work, we propose a new method, which couples a binary quantum-behaved particle swarm optimization with SVM approach, to select feature gene subset from cancer microarray data. In order to prove the advantages of BQPSO/SVM, we also implement two other algorithms, BPSO/SVM and GA/SVM. The BPSO and GA used in this work are both the original version. These two algorithms or improved ones were used in this case by other scholars early in [30–32]. All these three approaches are experimentally assessed on five well-known cancer data sets (Leukemia, Colon, Prostate, Lung, and Lymphoma).

This paper is structured as follows. In Section 2, we review the BQPSO algorithm, and in Section 3 the SVM technique is described and our BQPSO/SVM method is proposed. In Section 4, the five microarray data sets used in this work are described. Experimental results are presented in Section 5, including biological descriptions of several obtained genes. Finally, the paper is concluded in Section 6.

## 2. Binary Encoded Quantum-Behaved Particle Swarm Optimization (BQPSO)

PSO algorithm is a population-based evolutionary search technique, which was firstly proposed in [33]. Social behavior of animals such as bird flocking and fish schooling and swarm theory is the underlying motivation for the development of PSO. Inspired by the quantum theory, Sun et al. [34] developed a novel variant of PSO called Quantum-behaved Particle Swarm Optimization (QPSO), where a strategy based on a quantum *δ* potential well is employed to sample around the personal best points and then introduced the mean best position into the algorithm [35–37].

**Figure 1:**
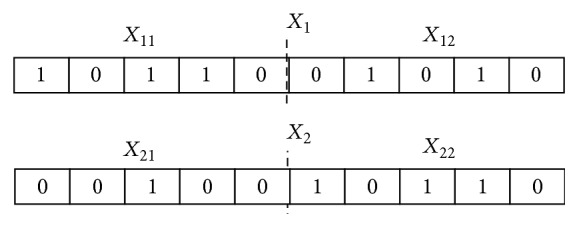
Binary coding of particle's position.

Based on our previous work in [38], in this paper, we further proposed a discrete binary version of QPSO (BQPSO) as a search algorithm coupling SVM for gene selection based on cancer gene expression data. In the proposed BQPSO, the position of the particle is represented as a binary string. For instance, in Figure 1 *X*_1_(1011001010) is the first particle and *X*_2_(0010010110) is the second one; they all have two substrings (two decision variables), and the distance is defined as the Hamming distance between two binary strings; namely,

(1) $$\begin{array}{c} \left|\;X_{1}-X_{2}\;\right|=d_{H}\left(\;X_{1},X_{2}\;\right), \end{array}$$

where *d*_*H*_(·) is the function to get Hamming distance between *X*_1_ and *X*_2_, which is the count of bits different in the two strings; the distance is seven in Figure 1. In the BQPSO, the dimension is defined as the number of decision variables, so that a particle can have more than one decision variable. For example, particle *i* is represented as *X*_*i*_ = (*X*_*i*1_, *X*_*i*2_,…, *X*_*iD*_), and it has *D* decision variables, and *X*_*id*_ refers to the *d*th substring (*d*th decision variable) of the position of the *i*th particle. Given that the lengths of *X*_*id*_ and *X*_*i*_ are *l*_*d*_ and *l*, respectively, then we can get equation as follows:

(2) $$\begin{array}{c} l=\overset{d}{\underset{i=1}{\sum }}l_{d},\;d=1,2,\ldots ,D. \end{array}$$

In the BQPSO, the mean best (*mbest*) position of all particles is determined by the states of the bits of all particles' *pbest*. In detail, for *j*th bit of the *mbest*, if 1 appears more often than 0 at the *j*th bit of all *pbest*, the *j*th bit of *mbest* will be 1; otherwise the bit will be 0. However, if 1 and 0 have the same frequency of occurrence, the *j*th bit of *mbest* will be set randomly to be 1 or 0, with probability 0.5 for either state. The function for obtaining *mbest* is called *mbest* = Get_*mbest* (*pbest*). The pseudocode of the function for obtaining *mbest* is given in Pseudocode 1.

*P*_*i*_ is the coordinate of local attractor for particle *i*. In the continuous version of QPSO, the coordinate *P*_*id*_ of *P*_*i*_ lies between *pbest*_*id*_ and *gbest*_*d*_. In the BQPSO, the point *P*_*i*_ is generated through one-point or multipoint crossover operation of *pbest*_*i*_ and *gbest* like that used in genetic algorithm (GA), and this definitely make *P*_*i*_ lay between *pbest*_*id*_ and *gbest*_*d*_ as well. The function getting *mbest* in BQPSO is called *P*_*i*_ = Get_*P* (*pbest*_*i*_, *gbest*).

Update equation of the particle position in the original QPSO is given by

(3) $$\begin{array}{c} \left|\;X_{id}-P_{id}\;\right|=\alpha \left|\;mbest_{d}-X_{id}\;\right|\ln\left(\;\frac{1}{\mu }\;\right),\;\mu =\mathrm{rand}\left(\right). \end{array}$$

In the BQPSO, (4) can be written again as follows:

(4) $$\begin{array}{c} d_{H}\left(\;X_{id},P_{id}\;\right)=\left\lceil \;b\;\right\rceil , \end{array}$$

where

(5) $$\begin{array}{c} b=\alpha *d_{H}\left(\;X_{id},mbest_{d}\;\right)*\ln\left(\;\frac{1}{\mu }\;\right),\;\;\mu =\mathrm{rand}\left(\right). \end{array}$$

Because *d*_*H*_(·) is Hamming distance, *b* must be an integer, which is the reason for the use of function ⌈·⌉. New string *X*_*i*_ is obtained by the mutation of *P*_*i*_ with the probability computed by

(6) $$\begin{array}{c} P_{r}=\left\{\begin{array}{cc} \frac{b}{l_{d}}, & \\ 1, & \text{if }\frac{b}{l_{d}}>1. \end{array}\right. \end{array}$$

In [35], here *l*_*d*_ is the length of substring *X*_*id*_. Function getting *X*_*id*_ is denoted as *X*_*id*_ = Transf (*P*_*id*_, *P*_*r*_). The transformation of Transf (*P*_*id*_, *P*_*r*_) is described in Pseudocode 2.

The BQPSO can be summarized as Get_*mbest* (*pbest*), Get_*P* (*pbest*_*i*_, *gbest*), and Transf (*P*_*id*_, *P*_*r*_).

## 3. Gene Selection and Classification by BQPSO/SVM

### 3.1. The SVM Classifier

Support vector machine proposed in [39] is a technique derived from statistical learning theory. It is widely used to classify points by assigning them to one of two disjoint half spaces [40, 41]. That is to say SVM carries out mainly a 2-class classification. For linearly separable data, SVM gets the hyperplane which maximizes the margin between the training samples and the class boundary. For nonlinearly separable cases, samples are mapped to a high dimensional space. In this space, such a separating hyperplane can be found. The assignment is conducted by way of a mechanism called the kernel function.

Theoretically, SVM is able to correctly classify any linearly separable data. Consider the data with two classes, which can be expressed as

(7) $$\begin{array}{c} \left(\;x_{i},y_{i}\;\right),\;i=1,2,\ldots ,l,\;\;x\in R^{n},\;\;y\in \left\{\pm 1\right\}, \end{array}$$

and then the hyperplane that separated the two classes of the data is given by

(8) $$\begin{array}{c} \left(\;w\cdot x\;\right)+b=0. \end{array}$$

In order to guarantee that the data can be correctly classified and the distance between the classes is as large as possible, the hyperplane must satisfy

(9) $$\begin{array}{c} y_{i}\left[\;\left(\;w\cdot x_{i}\;\right)+b\;\right]\geq 1,\;i=1,2,\ldots ,l, \end{array}$$

by which the distance is obtained as 2/‖*w*‖ so that the problem of constructing the hyperplane is converted to the following optimization problem:

(10) min ϕw=12w2=12w′·w

with (9) being the constraint. By introducing the following Lagrange function to solve problem (10):

(11) $$\begin{array}{c} L\left(\;w,b,a\;\right)=\frac{1}{2}\left\|\;w\;\right\|-a\left(\;y\left(\;\left(\;w\cdot x\;\right)+b\;\right)-1\;\right), \end{array}$$

where *a* > 0 is known as the Lagrange coefficient. Solving the Lagrangian dual of the problem, one obtains a simplified problem:

(12) max Qa=∑j=1laj−12∑i=1 l ∑j=1laiajyiyjxi·xjs.t. ∑j=1lajyj=0j=1,2,…,l,  aj≥0.

Solving the problem in (12), we can get

(13) a∗=a1∗,a2∗,…,al∗T,w∗=∑j=1laj∗yjxj,b∗=yi−∑j=1lyjaj∗xj·xi,j∈j ∣ aj∗≻0,

by which the hyperplane is obtained as

(14) $$\begin{array}{c} \left(\;w^{*}\cdot x\;\right)+b^{*}=0 \end{array}$$

and the optimal classification function is

(15) fxsgn⁡w∗·x+b∗=sgn⁡∑j=1laj∗yjxj·xi+b∗,x∈Rn.

### 3.2. The Proposed BQPSO/SVM Approach

In many bioinformatics problems the number of features is significantly larger than the number of samples. In order to improve the classification or to help to recognize interesting features in noisy environments, tools for reducing the number of features are indispensable. The hybrid BQPSO/SVM approach proposed in the following contributes especially in this sense.

First of all, the data should be preprocessed. Normalization of data must be conducted so as to eliminate the impact of the dimensionless on the classification. Then we need to take traditional *t*-test on the data, order the genes by *p* value ascending, and get 50 top-ranked genes from all. Afterwards, most of the noisy data have been removed. These 50 genes comprise the whole search space of the BQPSO algorithm for gene selection.

For the BQPSO used in this work, the swarm sizes for the BQPSO and BPSO were set to be 20 and the population size for GA was also 20. Each particle has just one decision variable, and thus the dimension of the particle is just one. The length of the particle is 50, so every particle is a binary string with length of 50, and 1 represents that this gene is chosen and 0 is not. Feature gene selection and cancer classification based on hybrid BQPSO/SVM algorithm can be described as the procedure in Pseudocode 3.

### 3.3. Evaluation Function

Since a particle *X*_*i*_ is a binary string representing a gene subset in BQPSO/SVM, the evaluation of each particle is executed by the SVM classifier to assess the quality of the represented gene subset. The fitness of a particle *X*_*i*_ is calculated employing a leave-one-out cross validation (LOOCV) method to calculate the accuracy of SVM trained with this subset. In leave-one-out cross validation, one of all samples is evaluated as test data while the others except this one are used as training data, repeated until all samples have been used as test data. The classification accuracy of LOOCV is the average accuracy of *n* times classifying, if the data set has *n* samples. The evaluation function is described in

(16) $$\begin{array}{c} fitness\left(\;X_{i}\;\right)=\alpha *accuracy+\beta *\frac{50}{feature\_number}, \end{array}$$

where *α* and *β* are weight values and set to 0.6 and 0.4, respectively, for the purpose of controlling that the accuracy value takes precedence over the subset size, since high accuracy is preferred when leading the search process. The target here consists of maximizing the accuracy and minimizing the number of genes (feature_number). For convenience (only maximum of fitness), the second factor is presented as 50/feature_number.

## 4. The Data Sets

There are several DNA microarray data sets from published cancer gene expression studies. Five of them were used in this paper. They are Leukemia data set, Prostate data set, Colon data set, Lung data set, and Lymphoma data set. All of them were taken from the BRB-ArrayTools in [42] with URL http://linus.nci.nih.gov/~brb/DataArchive_New.html. More details of these five data sets are showed in Tables 1 and 2. The value in parenthesis in Table 3 is the number of examples of class 1 or class 2 involved in that data set.

## 5. Experimental Results and Performance Comparison

BQPSO/SVM approach was implemented on MATLAB, along with BPSO/SVM and GA/SVM. The SVM classifier used in these three approaches is based on the LIBSVM library in [43]. For the SVM configuration, since we were considering the performance of the search algorithm in the work, rather than the influence of parameters in SVM to classification, we used the default parameters of LIBSVM. And the default kernel function was configured as radial basis function. The fitness function in this work is the classification accuracy of leave-one-out cross validation (LOOCV).

All experiments were carried out using a PC with Windows OS and a Pentium Dual-Core 2.60 GHz CPU, with 2 G of RAM. BQPSO/SVM, BPSO/SVM, and GA/SVM algorithms on five cancer related microarray data sets were independent executed 25 times over each data set, in order to have statistically meaningful conclusions as these three algorithms are stochastic search methods.

### 5.1. Parameter Settings

The parameters used in BQPSO, BPSO, and GA algorithms are shown in Table 3. These parameters were selected after several test evaluations of each algorithm and data set instance until reaching the best configuration in terms of the overall quality of solutions.

### 5.2. Discussion and Analysis

Depending on the results of the experiments, we made analysis of results focusing on the performance and robustness, as well as the quality of the obtained solutions providing a biological description of most significant ones. We conducted the experiments for BPSO/SVM and GA/SVM in order to demonstrate the advantage of the proposed BQPSO/SVM without any other factors affecting, since in our work all these three algorithms are operated in exactly the same hardware and software environment and with the same data sets and parameters.

#### 5.2.1. Performance Analysis

Next, we compare BQPSO/SVM with BPSO/SVM and GA/SVM. Since these three algorithms are running in the same environment, parameters, and data sets, the results are absolutely comparable.  Table 4 lists the highest LOOCV accuracy in 25 independent executions of each method for each data set. The mean columns contain the average of the LOOCV accuracy obtained from 25 independent executions.

The performance comparison shows that, compared to BPSO/SVM and GA/SVM, BQPSO/SVM has an obvious advantage. In terms of the correct rate, the search capability of BQPSO/SVM is stronger than the other two competitors.

**Figure 2:**
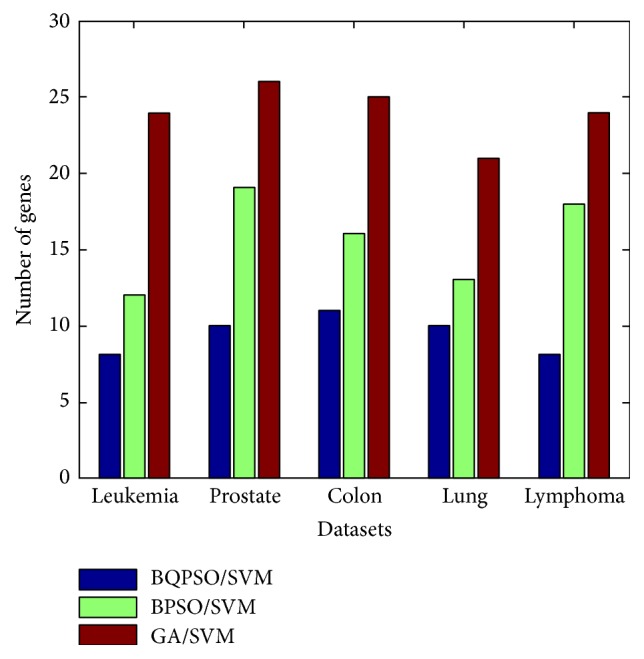
The average number of genes selected by BQPSO/SVM, BPSO/SVM, and GA/SVM, respectively.

The purpose of feature selection in our work is to find small subsets with high classification accuracy. In Figure 2, the number of genes is the mean size of subsets from 25 executions. Obviously, the proposed BQPSO/SVM provided smaller subsets of genes than the other two methods.

#### 5.2.2. Algorithm Robustness

Besides the quality of the algorithm, its ability to generate similar or identical results when executed several times is also important. One of the most important norms in assessing any proposed algorithm is robustness. It is particularly important for metaheuristics which are employed in this work. The standard deviation (std. dev.) in Table 5 denotes the standard deviation of accuracy from 25 independent executions. As it can be seen from the standard deviation, the robustness of the proposed algorithm is significantly better than GA/SVM. Compared with BPSO/SVM, our proposed algorithm obtained smaller standard deviation with Prostate data set and Colon data set but found much better solutions which led to a larger standard deviation. Overall, from Table 5, it is shown that BQPSO/SVM has an obvious advantage over the other two approaches in terms of robustness.

#### 5.2.3. Brief Biological Analysis of Selected Genes

Finally, the best subsets of genes were found for each data set. We add up all subsets having the highest accuracy and list the selected genes. For Colon data set, the top 5 genes with the highest selection frequency of each microarray data are presented in Table 6.

- Among the genes listed in Table 5, two of them were also selected by [44]. The first gene is uroguanylin precursor Z50753. It was shown that a reduction of uroguanylin might be an indication of colon tumors in [45, 46] which reported that treatment with uroguanylin has a positive therapeutic significance to the reduction in precancerous colon ploys.
- The second selected gene of colon data set is R87126 (myosin heavy chain, nonmuscle). The isoform B of R87126 serves as a tumor suppressor and is well known as a component of the cytoskeletal network [47].

## 6. Conclusion

In this paper, a hybrid technique for gene selection and classification of high dimensional DNA Microarray data was presented and compared. This technique is based on a metaheuristic algorithm BQPSO used for feature selection using the SVM classifier to identify potentially good gene subsets and is compared with the BPSO and GA. In addition, genes selected are validated by an accurate leave-one-out cross validation method to improve the actual classification.

All three approaches were experimentally assessed on five well-known cancer data sets. Results of 100% classification rate and less than average 11 genes are obtained in most of our executions. The use of preprocessing method has shown a great influence on the performance of proposed algorithm, since it introduces an early set of acceptable solutions in their evolution process. Continuing the line of this work, we are interested in optimization of BQPSO/SVM in order to discover new and better subsets of genes using specific Microarray data sets.

## Figures and Tables

**Pseudocode 1:**
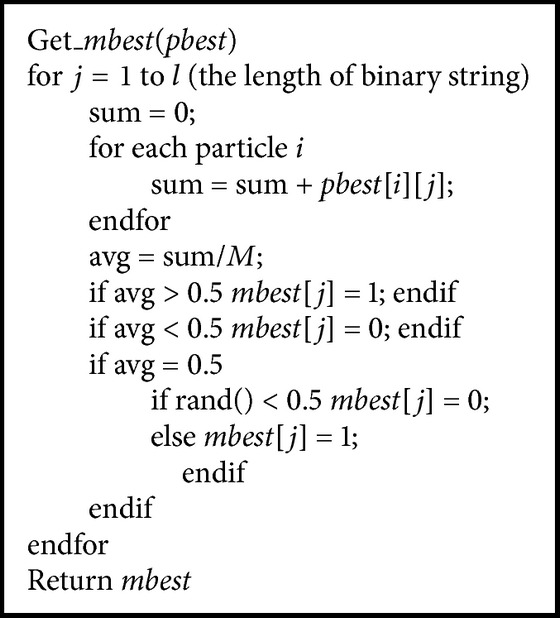
Pseudocode for obtaining *mbest*.

**Pseudocode 2:**
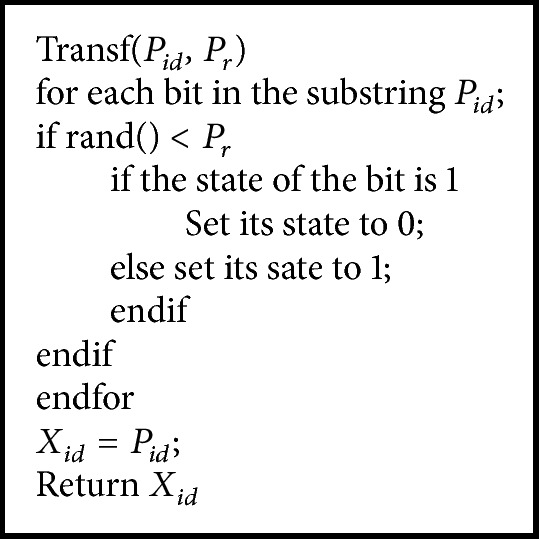
Pseudocode of the transformation.

**Pseudocode 3:**
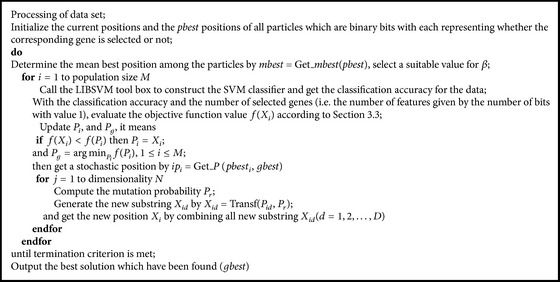
The psudocode the BPSO/SVM.

**Table 1:** Description for the test databases.

Number	Name of data set	Number of examples	Number of genes	Classes
1	Leukemia	72	7129	2
2	Prostate	102	12600	2
3	Colon	62	2000	2
4	Lung	181	12533	2
5	Lymphoma	77	7129	2

**Table 2:** Description for the test databases.

Number	Name of data set	Class 1 (quantity)	Class 2 (quantity)
1	Leukemia	AML (25)	ALL (47)
2	Prostate	N^1^ (50)	PC^2^ (52)
3	Colon	N^3^ (22)	CC^4^ (40)
4	Lung	MPM^5^ (31)	ADCA^6^ (150)
5	Lymphoma	DLBCL^7^ (58)	FL^8^ (19)

1: normal, 2: prostate cancer, 3: normal, 4: colon cancer, 5: malignant pleural mesothelioma, 6: adenocarcinoma, 7: diffuse large B-cell lymphoma, and 8: follicular lymphoma.

**Table 3:** BQPSO, BPSO, and GA parameters for gene subset selection and classification.

BQPSO	

Swarm size	20
Iteration	100
Dimension of particle	1
*β*	1

BPSO	

Swarm size	20
Iteration	100
Maximum of velocity	6
(*w*, *c*1, *c*2)	(0.5, 2, 2)

GA	

Swarm size	20
Iteration	100
Probability of crossover	0.9
Probability of mutation	0.04

**Table 4:** Comparison of accuracy with the proposed algorithm, BPSO/SVM, and GA/SVM.

Data set	BQPSO/SVM		BPSO/SVM		GA/SVM	
	Best	Mean	Best	Mean	Best	Mean
Leukemia	100	100	100	100	100	99.61
Prostate	100	99.25	99.02	99.02	98.04	96.00
Colon	93.55	92.52	91.94	91.94	91.94	88.65
Lung	100	99.96	100	99.96	100	99.87
Lymphoma	100	99.79	100	99.74	98.70	98.18

**Table 5:** Comparison in terms of statistical results of BQPSO/SVM, BPSO/SVM, and GA/SVM.

Data set	BQPSO/SVM		BPSO/SVM		GA/SVM	
	Best	Std. dev.	Best	Std. dev.	Best	Std. dev.
Leukemia	100	0	100	0	100	0.64
Prostate	100	0.43	99.02	0	98.04	1.20
Colon	93.55	0.79	91.94	0	91.94	1.89
Lung	100	0.15	100	0.15	100	0.24
Lymphoma	100	0.49	100	0.53	98.70	0.75

**Table 6:** Top 5 genes with the highest selection frequency of colon data set.

Data set	Accession number	Gene description
Colon	Z50753	*H. sapiens *mRNA for GCAP-II/uroguanylin precursor
	R87126	Myosin heavy chain, nonmuscle (*Gallus gallus*)
	X63629	*H. sapiens* mRNA for p cadherin
	M76378	Human cysteine-rich protein (CRP) gene, exons 5 and 6
	X53586	Human mRNA for integrin alpha 6
